# Upregulation of Glutamic-Oxaloacetic Transaminase 1 Predicts Poor Prognosis in Acute Myeloid Leukemia

**DOI:** 10.3389/fonc.2020.00379

**Published:** 2020-03-24

**Authors:** Zhiheng Cheng, Yifeng Dai, Tiansheng Zeng, Yan Liu, Longzhen Cui, Tingting Qian, Chaozeng Si, Wenhui Huang, Ying Pang, Xu Ye, Jinlong Shi, Lin Fu

**Affiliations:** ^1^Department of Hematology, The Second Affiliated Hospital of Guangzhou Medical University, Guangzhou, China; ^2^Department of Pathology and Medical Biology, University of Groningen, University Medical Center Groningen, Groningen, Netherlands; ^3^Translational Medicine Center, State Key Laboratory of Respiratory Disease, The Second Affiliated Hospital of Guangzhou Medical University, Guangzhou, China; ^4^Department of Biomedical Sciences, University of Sassari, Sassari, Italy; ^5^Translational Medicine Center, Huaihe Hospital of Henan University, Kaifeng, China; ^6^Department of Operations and Information Management, China-Japan Friendship Hospital, Beijing, China; ^7^Department of Biomedical Engineering, Chinese PLA General Hospital, Beijing, China; ^8^Department of Hematology, Huaihe Hospital of Henan University, Kaifeng, China

**Keywords:** acute myeloid leukemia, glutamate oxaloacetate transaminase 1, chemotherapy, allogeneic hematopoietic stem cell transplantation, prognosis

## Abstract

One of the key features of acute myeloid leukemia (AML), a group of very aggressive myeloid malignancies, is their strikingly heterogenous outcomes. Accurate biomarkers are needed to improve prognostic assessment. Glutamate oxaloacetate transaminase 1 (GOT1) is essential for cell proliferation and apoptosis by regulating cell's metabolic dependency on glucose. It is unclear whether the expression level of *GOT1* has clinical implications in AML. Therefore, we analyzed the data of 155 AML patients with *GOT1* expression information from The Cancer Genome Atlas (TCGA) database. Among them, 84 patients were treated with chemotherapy alone, while 71 received allogeneic hematopoietic stem cell transplantation (allo-HSCT). In both treatment groups, high *GOT1* expression was associated with shorter event-free survival (EFS) and overall survival (OS) (all *P* < 0.05). Multivariate analysis identified several independent risk factors for both EFS and OS in the chemotherapy-only group, including high *GOT1* expression, age ≥60 years, white blood cell count ≥15 × 10^9^/L, bone marrow blasts ≥70%, and *DNMT3A, RUNX1* or *TP53* mutations (all *P* < 0.05); but in the allo-HSCT group, the only independent risk factor for survival was high *GOT1* expression (*P* < 0.05 for both EFS and OS). Kyoto Encyclopedia of Genes and Genomes (KEGG) enrichment analysis showed that the genes related to *GOT1* expression were mainly concentrated in “hematopoietic cell lineage” and “leukocyte transendothelial migration” signaling pathways. Collectively, *GOT1* expression may be a useful prognostic indicator in AML, especially in patients who have undergone allo-HSCT.

## Introduction

Acute myeloid leukemia (AML), the most common type of acute leukemia, is a clinically, genetically, and molecularly heterogeneous disease associated with uncontrolled proliferation and blocked differentiation of immature myeloid progenitors (1). The choice of postremission strategy depends on AML risk stratification. The European LeukemiaNet (ELN) system is the most widely accepted risk stratification system in the postgenomic era. It heavily utilizes cytogenetic and molecular aberrations, such as *FLT3-ITD, NPM1, CEBPA, RUNX1, TP53*, and *ASXL1* mutations (2). Additionally, abnormal oncogene expressions have attracted more attention in recent years, with great potentials to be incorporated into a refined AML risk stratification system (3, 4). For example, high expressions of the secreted frizzled-related protein 2 (*sFRP2*) and docking protein 7 (*DOK7*) genes are associated with better outcome (5, 6), while high expressions of *FHL2* and *iASPP* are associated with poor survival in AML (7).

Despite the improvements in AML prognostication, individual outcomes within each risk group, especially the intermediate group, are still markedly heterogenous (8). Deeper understanding of leukemogenesis may inspire the identification of biomarkers that can allow molecular-based classification and risk-adapted therapies to improve the outcome of AML. Reprogrammed cellular metabolism is a hallmark of cancer (9). In pancreatic cancer (PC) cells and colorectal cancer (CRC) cells, a defect in an electron transport chain will convert the cells to depend on glutamine as the major energy source for cell growth and proliferation (10, 11). Upregulation of glutamate oxaloacetate transaminase 1 (GOT1), an essential and ubiquitous enzyme in glutamine metabolism, is present in many types of human cancer and correlates with poor prognosis (12, 13). A study shows that incubation of *KRAS* mutant CRC cells with GOT1 promotes proliferation and reduces apoptosis, suggesting that GOT1 is required for cell survival (14). PC cells are notoriously sensitive to glutamine deficiency because glutamine keeps their cellular redox state, that they rely on GOT1 to reprogram their glutamine metabolism; knocking down *GOT1* can reduce PC cells' viability (15, 16).

Currently, the clinical and prognostic relevance of GOT1 in AML is unclear. Whether aberrant *GOT1* expression could alter the effect of allogeneic hematopoietic stem cell transplantation (allo-HSCT), a curative treatment for AML (17), is also unanswered. Hence, we conducted a biomarker study to evaluate the clinical and prognostic value of GOT1 in AML, and whether allo-HSCT can overcome its prognostic effect.

## Materials and Methods

### Patients

The Cancer Genome Atlas (TCGA) database (https://cancergenome.nih.gov/) were screened for patients with *GOT1* expression data and a total of 155 AML patients were included in this study (18). Peripheral blood samples were collected from all patients before treatment, and the Affymetrix microarray (U133) was used to analysis the expression of *GOT1*. Eighty-four patients were given chemotherapy alone, and were defined as the chemotherapy-only group; the other 71 patients with poor-risk features received allo-HSCT, and were defined as the allo-HSCT group. In all patients, age ranged from 18 to 88. Clinical and molecular characteristics at diagnosis were publicly accessible through TCGA database, such as peripheral blood (PB) white blood cell count (WBC), PB and bone marrow (BM) blast percentage, French-American-British (FAB) subtypes, and the frequencies of known recurrent genetic mutations. Primary endpoints were event-free survival (EFS) and overall survival (OS). The former was defined as the time from diagnosis to the first event including relapse, death, failure to achieve complete remission, or was censored at the last follow up. The latter was the time from diagnosis to death from any cause, or was censored at the last follow-up.

### Statistical Analysis

Descriptive statistics were used to summarize patients' clinical and molecular characteristics by median and/or range. Due to the non-normal distribution of the numerical data, a nonparametric test, i.e., the Wilcoxon-Mann-Whitney test, was used for between-group comparison. The chi-square test was used for between-group comparison of categorical data. Survival was estimated using the Kaplan-Meier methods and log-rank test. For multivariate analysis of EFS and OS, Cox proportional hazard models were constructed, using a limited backward elimination procedure. Spearman correlation analysis was used to determine the relationships between *GOT1* expression and genome expression profile. Multiple testing errors were assessed by false discovery rate (FDR). Kyoto Encyclopedia of Genes and Genomes (KEGG) enrichment analysis was conducted to evaluate the enrichment of *GOT1*-related gene expressions. A two-tailed *P* < 0.05 was considered statistically significant. All statistical analyses were performed by the SPSS software 22.0, the R software 3.5.0, and the GraphPad Prism software 7.0.

## Results

### Differences in Clinical and Molecular Characteristics Between *GOT1*^high^ and *GOT1*^low^ Patients in Different Treatment Groups

The chemotherapy-only and the allo-HSCT groups were each divided in two by the respective median *GOT1* expression levels. Within each group, the comparison of clinical and molecular characteristics between high and low *GOT1* expression subgroups were summarized in Table 1.

**Table 1:** Clinical and molecular characteristics of patients in different treatment groups.

**Characteristics**	**Chemotherapy-only group**			**Allo-HSCT group**		
	**High GOT1**	**Low GOT1**	***P***	**High GOT1**	**Low GOT1**	***P***
	**(*n* = 42)**	**(*n* = 42)**		**(*n* = 35)**	**(*n* = 36)**	
Age/years, median (range)	64 (33–82)	67 (22–88)	0.431*	51 (22–65)	53 (18–72)	0.366*
Age group/*n* (%)			1.000^§^			0.464^§^
<60 years	13 (31.0)	13 (31.0)		27 (77.1)	25 (69.4)	
≥60 years	29 (69.0)	29 (69.0)		8 (22.9)	11 (30.6)	
Gender/*n* (%)			0.274^§^			0.390^§^
Male	20 (47.6)	25 (59.5)		22 (62.9)	19 (52.8)	
Female	22 (52.4)	17 (40.5)		13 (37.1)	17 (47.2)	
WBC/ × 10^9^/L, median (range)	14.1 (1.4–297.4)	14.7 (0.7–171.9)	0.925*	30.5 (0.6–223.8)	29.4 (0.8–202.7)	0.538*
BM blast/%, median (range)	73 (30–99)	70 (32–98)	0.626*	72 (30–100)	64 (39–99)	0.527*
PB blast/%, median (range)	27 (0–98)	22 (0–97)	0.473*	54 (0–91)	40 (0–96)	0.341*
**FAB subtypes/*****n*** **(%)**						
M0	1 (2.4)	6 (14.3)	0.109^§^	2 (5.7)	7 (19.4)	0.151^§^
M1	10 (23.8)	10 (23.8)	1.000^§^	15 (42.9)	8 (22.2)	0.063^§^
M2	13 (31.0)	8 (19.0)	0.208^§^	11 (31.4)	7 (19.4)	0.246^§^
M3	0 (0.0)	0 (0.0)	1.000^§^	0 (0.0)	1 (2.8)	0.493^§^
M4	9 (21.4)	11 (26.2)	0.608^§^	4 (11.4)	9 (25.0)	0.139^§^
M5	6 (14.3)	6 (14.3)	1.000^§^	2 (5.7)	2 (5.6)	1.000^§^
M6	1 (2.4)	0 (0.0)	1.000^§^	1 (2.9)	0 (0.0)	0.493^§^
M7	2 (4.8)	1 (2.4)	1.000^§^	0 (0.0)	2 (5.6)	0.493^§^
**Cytogenetics/*****n*** **(%)**						
Normal	23 (54.8)	17 (40.5)	0.190^§^	23 (65.7)	10 (27.8)	0.001^§^
Complex karyotype	4 (9.5)	7 (16.7)	0.332^§^	3 (8.6)	8 (22.2)	0.112^§^
8 Trisomy	0 (0.0)	0 (0.0)	1.000^§^	2 (5.7)	4 (11.1)	0.674^§^
inv(16)/CBFβ-MYH11	0 (0.0)	6 (14.3)	0.026^§^	0 (0.0)	5 (13.9)	0.054^§^
11q23/MLL	3 (7.1)	0 (0.0)	0.241^§^	1 (2.9)	2 (5.6)	1.000^§^
−7/7q-	1 (2.4)	2 (4.8)	1.000^§^	2 (5.7)	1 (2.8)	0.614^§^
*t*_(15, 17)_/PML-RARA	0 (0.0)	0 (0.0)	1.000^§^	0 (0.0)	1 (2.8)	1.000^§^
*t*_(9, 22)_/BCR-ABL1	1 (2.4)	0 (0.0)	1.000^§^	1 (2.9)	1 (2.8)	1.000^§^
*t*_(8, 21)_/RUNX1-RUNX1T1	3 (7.1)	3 (7.1)	1.000^§^	0 (0.0)	1 (2.8)	1.000^§^
Others	7 (16.7)	7 (16.7)	1.000^§^	3 (8.6)	3 (8.3)	1.000^§^
**Risk/*****n*** **(%)**						
Good	4 (9.5)	9 (21.4)	0.074^§^	0 (0.0)	7 (19.4)	0.011^§^
Intermediate	23 (54.8)	23 (54.8)	0.803^§^	25 (73.5)	15 (41.7)	0.007^§^
Poor	15 (35.7)	10 (23.8)	0.266^§^	9 (26.5)	14 (38.9)	0.269^§^
*FLT3-ITD*/*n* (%)			0.002^§^			0.002^§^
Positive	13 (31.0)	2 (4.8)		14 (40.0)	3 (8.3)	
Negative	29 (69.0)	40 (95.2)		21 (60.0)	33 (91.7)	
*NPM1*/*n* (%)			0.002^§^			0.024^§^
Mutation	20 (47.6)	7 (16.7)		13 (37.1)	5 (13.9)	
Wild type	22 (52.4)	35 (83.3)		22 (62.9)	31 (86.1)	
*DNMT3A*/*n* (%)			0.221^§^			0.730^§^
Mutation	14 (33.3)	9 (21.4)		9 (25.7)	8 (22.2)	
Wild type	28 (66.7)	33 (78.6)		26 (74.3)	28 (77.8)	
*IDH1*/*IDH2*/*n* (%)			0.154^§^			0.832^§^
Mutation	5 (11.9)	10 (23.8)		8 (22.9)	9 (25.0)	
Wild type	37 (88.1)	32 (76.2)		27 (77.1)	27 (75.0)	
*RUNX1*/*n* (%)			0.019^§^			1.000^§^
Mutation	3 (7.1)	11 (26.2)		4 (11.4)	4 (11.1)	
Wild type	39 (92.9)	31 (73.8)		31 (88.6)	32 (88.9)	
*NRAS*/*KRAS*/*n* (%)			1.000^§^			0.710^§^
Mutation	6 (14.3)	6 (14.3)		4 (11.4)	3 (8.3)	
Wild type	36 (85.7)	36 (85.7)		31 (88.6)	33 (91.7)	
*TET2*/*n* (%)			0.332^§^			1.000^§^
Mutation	4 (9.5)	7 (16.7)		2 (5.7)	2 (5.6)	
Wild type	38 (90.5)	35 (83.3)		33 (94.3)	34 (94.4)	
*TP53*/*n* (%)			0.332^§^			1.000^§^
Mutation	4 (9.5)	7 (16.7)		2 (5.7)	2 (5.6)	
Wild type	38 (90.5)	35 (83.3)		33 (94.3)	34 (94.4)	
*MLL*/*n* (%)			0.004^§^			0.054^§^
Positive	10 (23.8)	1 (2.4)		4 (11.4)	0 (0.0)	
Negative	32 (76.2)	41 (97.6)		31 (88.6)	36 (100)	
Relapse/*n* (%)			0.821^§^			0.003^§^
Yes	16 (38.1)	15 (35.7)		29 (82.9)	18 (50.0)	
No	26 (61.9)	27 (64.3)		6 (17.1)	18 (50.0)	

WBC, white blood cell; BM, bone marrow; PB, peripheral blood; FAB, French American British; “*” denotes Mann-Whitney U test; “^§^” denotes chi-square test.

In the chemotherapy-only group, the *GOT1*^high^ subgroup had fewer patients with *CBF*β*-MYH11* (*P* = 0.026) and *RUNX1* mutations (*P* = 0.019), but more with *MLL* fusions (*P* = 0.004), *FLT3-ITD* and *NPM1* mutations (*P* = 0.002, *P* = 0.002, respectively), than the *GOT1*^low^ subgroup. Age, gender distribution, WBC count, BM/PB blast percentage, FAB subtype distribution, risk stratification, frequencies of other recurrent genetic mutations (*DNMT3A, IDH1*/*IDH2, NRAS*/*KRAS, TET2*, and *TP53*), as well as the relapse rates, were similar in the two subgroups.

In the allo-HSCT group, normal karyotype (*P* = 0.001), intermediate-risk (*P* = 0.007), *FLT3-ITD* and *NPM1* mutations (*P* = 0.002, *P* = 0.024, respectively) were more common in the *GOT1*^high^ subgroup, but *CBF*β*-MYH11* (*P* = 0.054) and good-risk (*P* = 0.011) were less in the *GOT1*^high^ subgroup. They also had higher relapse rate than the *GOT1*^low^ subgroup. Age, gender distribution, WBC count, BM/PB blast percentage, FAB distribution, and frequencies of other recurrent genetic mutations (*DNMT3A, IDH1*/*IDH2, RUNX1, NRAS*/*KRAS, TET2, TP53*, and *MLL*), were similar in the two groups.

### Prognostic Significance of *GOT1* Expression in AML

**Figure 1:**
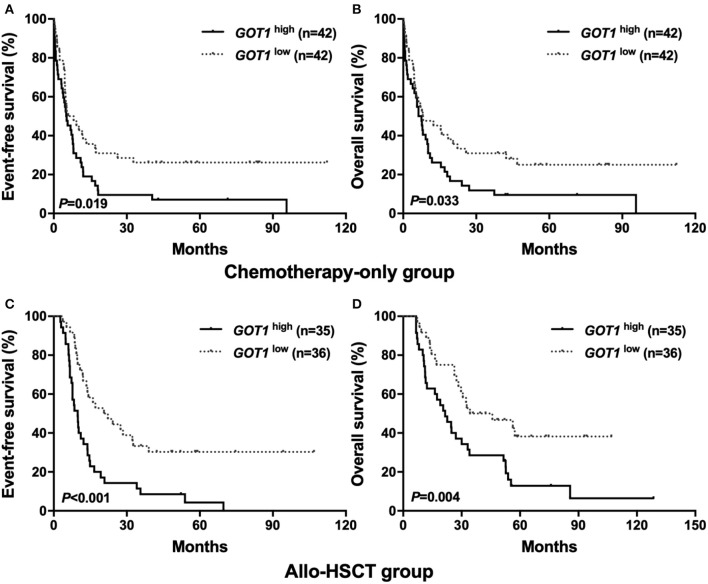
Kaplan-Meier curves of event-free survival (EFS) and overall survival (OS) in the chemotherapy-only and allo-HSCT groups. **(A,B)** In the chemotherapy-only group, high *GOT1* expressers had shorter EFS and OS than the low expressers. **(C,D)** In the allo-HSCT group, high *GOT1* expressers had shorter EFS and OS than the low expressers.

Using the Kaplan-Meier method, it was shown that in the chemotherapy-only group, the *GOT1*^high^ subgroup had shorter EFS and OS than their counterparts (*P* = 0.019, *P* = 0.033, Figures 1A,B); similar trend was also observed in the allo-HSCT group, where high *GOT1* expressers also had significantly worse survival than the low expressers (*P* < 0.001 for EFS, *P* = 0.004 for OS, Figures 1C,D).

### Possible Independent Prognostic Factors for the Two Groups

To further assess the prognostic value of *GOT1* in each group, the expression level of *GOT1* (high vs. low) and other commonly utilized AML risk stratification indices were used to construct multivariate analyses. The later included age (≥60 vs. <60 years), WBC count (≥15 vs. <15 × 10^9^/L), BM blast percentage (≥70 vs. <70%), PB blast percentage (≥20 vs. <20%), *FLT3-ITD* (positive vs. negative), and other common genetic mutations (*NPM1, DNMT3A, RUNX1, TET2*, and *TP53*; mutated vs. wild).

In the chemotherapy-only group (Table 2), high *GOT1* expression, older age, higher WBC count and BM blast percentage, and mutations in *DNMT3A, RUNX1*, and *TP53*, were independent risk factors for both EFS and OS (all *P* < 0.05).

**Table 2:** Multivariate analysis of EFS and OS in the chemotherapy-only group.

**Variables**	**EFS**		**OS**	
	**HR (95% CI)**	***P*-value**	**HR (95% CI)**	***P*-value**
*GOT1* (high vs. Low)	2.527 (1.470–4.344)	0.001	2.354 (1.353–4.097)	0.002
Age (≥60 vs. <60 years)	2.919 (1.564–5.448)	0.001	2.587 (1.372–4.880)	0.003
WBC (≥15 vs. <15 × 10^9^/L)	2.112 (1.130–3.948)	0.019	1.903 (1.050–3.450)	0.034
BM blasts (≥70 vs. <70%)	2.377 (1.362–4.149)	0.002	2.289 (1.311–3.999)	0.004
PB blasts (≥20 vs. <20%)	0.925 (0.531–1.610)	0.782	0.907 (0.515–1.597)	0.734
*FLT3-ITD* (positive vs. negative)	0.830 (0.406–1.696)	0.610	0.805 (0.381–1.703)	0.571
*NPM1* (mutated vs. wild)	0.836 (0.422–1.658)	0.608	0.647 (0.321–1.304)	0.223
*DNMT3A* (mutated vs. wild)	2.198 (1.205–4.011)	0.010	2.210 (1.226–3.984)	0.008
*RUNX1* (mutated vs. wild)	3.053 (1.405–6.636)	0.005	3.003 (1.417–6.365)	0.004
*TET2* (mutated vs. wild)	1.399 (0.606–3.230)	0.432	0.930 (0.420–2.062)	0.859
*TP53* (mutated vs. wild)	3.789 (1.677–8.563)	0.001	2.980 (1.361–6.526)	0.006

*EFS, Event-free survival; OS, Overall survival; HR, hazard ratio; CI, confidence interval; WBC, white blood cell; BM, bone marrow; PB, peripheral blood*.

In the allo-HSCT group (Table 3), high *GOT1* expression was also an independent risk factor for both EFS and OS (all *P* < 0.01). For EFS, other independent risk factors included higher WBC count, *FLT3-ITD* and *NPM1* mutations (all *P* < 0.05); *RUNX1* and *TP53* mutations were independent risk factors for OS (all *P* < 0.05).

**Table 3:** Multivariate analysis of EFS and OS in the allo-HSCT group.

**Variables**	**EFS**		**OS**	
	**HR (95%CI)**	***P*-value**	**HR (95%CI)**	***P*-value**
*GOT1* (high vs. Low)	3.444 (1.886–6.289)	0.000	2.470 (1.342–4.543)	0.004
Age (≥60 vs. <60 years)	1.132 (0.549–2.334)	0.737	1.364 (0.681–2.731)	0.381
WBC (≥15 vs. <15 × 10^9^/L)	2.242 (1.160–4.333)	0.016	1.339 (0.681–2.634)	0.398
BM blasts (≥70 vs. <70%)	1.088 (0.563–2.100)	0.802	1.005 (0.486–2.075)	0.990
PB blasts (≥20 vs. <20%)	1.065 (0.532–2.132)	0.859	1.488 (0.692–3.198)	0.308
*FLT3-ITD* (positive vs. negative)	2.092 (1.018–4.299)	0.045	2.172 (0.958–4.922)	0.063
*NPM1* (mutated vs. wild)	0.363 (0.163–0.808)	0.013	0.498 (0.209–1.184)	0.115
*DNMT3A* (mutated vs. wild)	1.465 (0.748–2.871)	0.266	1.730 (0.876–3.417)	0.115
*RUNX1* (mutated vs. wild)	1.508 (0.660–3.447)	0.330	2.979 (1.235–7.188)	0.015
*TET2* (mutated vs. wild)	0.429 (0.118–1.560)	0.199	0.474 (0.120–1.873)	0.287
*TP53* (mutated vs. wild)	3.171 (0.922–10.900)	0.067	10.362 (2.660–40.37)	0.001

*EFS, Event-free survival; OS, Overall survival; HR, hazard ratio; CI, confidence interval; WBC, white blood cell; BM, bone marrow; PB, peripheral blood*.

### Association Between Genome Expression Profile and *GOT1* Expression

**Figure 2:**
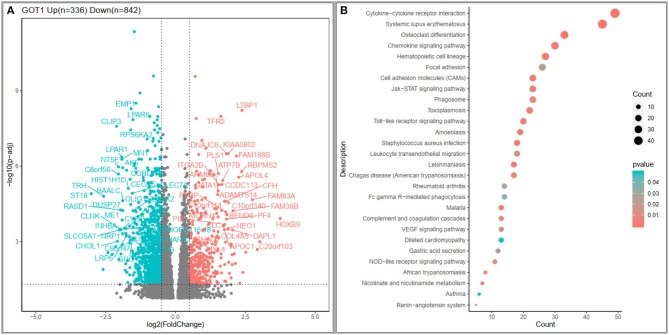
Genome expression profile and cell signaling pathways associated with *GOT1* expression. **(A)** Volcano plot of differential gene expression. Up-regulated and down-regulated genes were labeled with red and cyan dots, respectively. **(B)** Kyoto Encyclopedia of Genes and Genomes (KEGG) enrichment analysis of genes related to *GOT1* expression.

To explore possible clues to the effects of *GOT1* on AML, the high throughput sequencing information from TCGA database was used to summarize the *GOT1*-related gene expression profile. Three hundred and thirty-six up-regulated and 842 down-regulated genes that were significantly associated with *GOT1* expression (*P* < 0.05, Figure 2A) were screened. Furthermore, KEGG enrichment analysis indicated that the genes associated with *GOT1* expression were mainly concentrated in “cytokine-cytokine receptor interaction”, “osteoclast differentiation”, “chemokine signaling pathway”, “hematopoietic cell lineage”, and “leukocyte transendothelial migration” signaling pathways (Figure 2B).

### Discussion

In this retrospective study, we found that overexpression of *GOT1* is uniformly associated with poor survival in AML patients, regardless of the treatment modality they underwent; allo-HSCT might not abate its strong, detrimental effect on AML prognosis.

While both are vital nutrients, unlike glucose, glutamine can supply the cancer cells with both carbon and nitrogen (11). Not only is glutamine a strong growth signal, it also has important metabolites such as glucosamine, nucleic acids, and non-essential amino acids (NEAAs) (19)—all these metabolic processes require the activity of GOT1 (20). Based on our analyses, high expression of *GOT1* is more likely to co-exist with *FLT3-ITD* and *NPM1* mutations, and high *GOT1* expressers more frequently have worse outcomes. This suggests that there may be a superposition effect between the upregulation of *GOT1* and some adverse prognostic factors in AML.

The tumorigenesis role of *GOT1* has been studied in pancreatic cancer. A study demonstrated that one of the mechanisms of *KRAS* in inducing pancreatic ductal adenocarcinoma is by up-regulating *GOT1* and inhibiting glutamate dehydrogenase 1 (*GLUD1*), thus reprogramming glutamine metabolism (11). Furthermore, other studies indicated that non-canonical anaplerotic glutamine metabolism plays a significant role in the generation of nicotinamide adenine dinucleotide phosphate (NADPH); down-regulation of *GOT1* in pancreatic cancer cells could impair glutamine-dependent NADPH production, so as to stop cell growth (21). This metabolic process is also important in reducing reactive oxygen species (ROS) by coupling with other redox balance pathways such as glutathione synthesis (22). We found that high *GOT1* expression is an independent poor prognostic factor for AML patients. KEGG enrichment analysis demonstrated that genes (*LTBP1, TFR2, HOXB9, NEO1, DAPL1, EMP1, LPAR6, CLIP3, NRP1, SLCO5A1*, and *RPL5*) involved in “cytokine-cytokine receptor interaction”, “osteoclast differentiation”, “chemokine signaling pathway”, “hematopoietic cell lineage”, and “leukocyte transendothelial migration” signaling pathways are significantly correlated with the *GOT1* expression. It could be deduced that *GOT1* might be involved in leukemogenesis through aforesaid pathways, although this would require further studies to confirm.

Multivariate analysis indicated that in the chemotherapy-only group, age ≥60 years, BM blasts ≥70%, and WBC count ≥15 × 10^9^/L also independently contribute to poor survival. This is concordant with older studies that older age confers unfavorable prognosis in AML, possibly due to higher mutation burden, lower baseline performance status and more co-morbidities in this population (23). The deleterious effects of highly-active bone marrow blast proliferation and high peripheral WBC count on AML remission rate and survival are also well described in previous findings that they have adverse effect on OS (24, 25). In the chemotherapy-only group, *DNMT3A, TP53*, and *RUNX1* mutations are also independent risk factors for EFS and OS, consistent with previous studies (26–28). But in the allo-HSCT group, apart from *GOT1*, only *TP53* and *RUNX1* mutations are associated with inferior OS. We speculated that allo-HSCT could reverse the adverse effects of *FLT3-ITD, NPM1*, and *DNMT3A* mutations, but could not reverse the impact of *GOT1* expression. *GOT1* might be a better prognostic biomarker than the other widely-used molecular markers.

In summary, our findings show that high *GOT1* ex*p*ression is an independent poor prognostic biomarker in AML, and its adverse prognostic effect couldn't be overcome by allo-HSCT. Given the relative simplicity to measure *GOT1* expression at diagnosis and its distinct prognostic value, it is reasonable to envision it as a biomarker for risk stratification and guidance for treatments in AML. Our study was limited by its small, retrospective nature, and the results would need to be verified in a larger, prospective cohort.

## Data Availability Statement

All data in this study were publicly accessible from The Cancer Genome Atlas (TCGA) database (https://cancergenome.nih.gov/). We were not involved direct interaction with patients. All analyses during this study were included in this article.

## Author Contributions

ZC and LF conceived the study and wrote the manuscript. YD and TZ collected and analyzed the data. YL, LC, TQ, CS, WH, YP, XY, and JS participated in analyzing and discussing the results. All authors edited and approved the final manuscript.

### Conflict of Interest

The authors declare that the research was conducted in the absence of any commercial or financial relationships that could be construed as a potential conflict of interest.
